# Influenza Revisited

**DOI:** 10.3201/eid1201.051442

**Published:** 2006-01

**Authors:** Jeffery K. Taubenberger, David M. Morens

**Affiliations:** * Armed Forces Institute of Pathology, Rockville, Maryland, USA;; † National Institutes of Health, Bethesda, Maryland, USA

**Keywords:** Influenza, pandemic influenza, epidemic influenza, overview

This issue of Emerging Infectious Diseases includes a group of invited articles addressing pandemic influenza. Over the past 2 years, concerns about a new influenza pandemic caused by either an epizootic avian strain, such as H5N1, or by some other influenza virus have engaged top virologists, epidemiologists, and policymakers as well as the press and public (*1**,**2*). However, many scientific questions about the risk of a pandemic remain unanswered, and as science attempts to catch up on decades of relative neglect, fear and speculation have begun to mount. Such speculation has led to what the press has called "hysteria" in private stockpiling of antiviral drugs; this panic has even been compared to the widespread fear of an atomic bomb attack that gripped the United States in the late 1950s and early 1960s, when many citizens built and stocked underground fallout shelters.

In this climate, scientific and public health communities must continually update and review what is known about the risk of pandemic influenza and about options to prevent and control it. This group of articles is intended to serve as a modest database of current knowledge and informed opinion in several key areas, including the history of pandemic influenza and public health responses to it; influenza pathogenesis, natural history, and host immune responses to infection; and influenza prevention and treatment with drugs and vaccines.

Missing from the list of authors in this issue is a man whose insight, effort, and support probably did more to advance our understanding of influenza than the efforts of any other single individual over the past 30 years, John R. LaMontagne, whose untimely death in 2004 was a great loss to the scientific community (for additional information, see http://www3.niaid.nih.gov/about/overview/previousdirectors/LaMontagne/).

John would have agreed with another visionary scientist, Hermann Pidoux (1808–1882), who observed that "epidemics are the lives of diseases." In an attempt to understand a disease as explosive and fatal as pandemic influenza, the classic emerging/reemerging infectious disease, its history has been self-consciously chronicled for several centuries. The importance of that effort was recognized during the pandemic of 1889 and strongly reinforced by the next pandemic in 1918–1919 (the so-called "Spanish flu," the deadliest pandemic in human history). We review the life cycle of pandemic influenza during the past century, including the pandemics of 1918, 1957, 1968, and 1977, as well as a feared nonpandemic in 1976, looking at pandemics from different angles, questioning whether they are predictable and, if they are, what telltale signs we should be looking for.

The answers to these questions may not be reassuring. The origin of the earliest human influenza virus yet identified, the 1918 pandemic virus, is still a mystery even after genetic sequencing and comparison with other historical and circulating influenza viruses (*3**,**4*). Though clearly descended from an avian virus, the 1918 strain is genetically unlike any other influenza virus examined over the past 88 years, which indicates that its immediate origin before the pandemic is an unknown source. Complicating problems of origin, all of the pandemic and epidemic influenza A viruses that have appeared since 1918 have been descendants of it, arising by either genetic drift, reassortment with prevalent avian viruses, or in 1 case (1977) by apparent release from a freezer. Thus, little scientific basis exists for predicting whether the current enzootic/epizootic avian H5N1 virus will become pandemic: none of the known pandemic influenza events of the past 87 years seem to have much in common with the current H5N1 situation.

Another problem is learning about the mechanisms by which influenza A viruses, all of which are believed to be endemic in wild waterfowl, their natural hosts, acquire the capacities to switch hosts, produce diseases in these new hosts, and in some cases, establish the ability to propagate directly between them. While preliminary information about the first 2 of these capacities is gradually becoming known (*5**–**7*), little has been learned about the third. Thus, predicting whether current H5N1 viruses are moving in the direction of solving the ultimate challenge of host-switching/propagation in humans, or whether they are fundamentally incapable of doing so, is difficult.

Although science may yet offer little in the way of pandemic prediction, understanding the size of the influenza problem and the mechanisms by which influenza viruses cause severe and fatal disease, i.e., pathogenesis, is still important. Such knowledge is fundamental if we expect to prevent and control epidemics using public health measures and clinical therapies. Again, answers are elusive. Although influenza is a leading cause of death worldwide, measuring the total effect of deaths from influenza is impossible, in part because diagnostic records for a key risk group, the elderly, are incomplete and imprecise (*8*). Influenza also kills by different mechanisms such as primary viral pneumonia, secondary bacterial pneumonia in virus-damaged lungs, and an acute respiratory distresslike syndrome possibly associated with overly brisk immune responses, as well as by cardiac and other pathways, and by exacerbating serious chronic diseases such as diabetes mellitus, renal diseases, and congestive heart failure. The problems of understanding influenza occurrence and pathogenesis are therefore complicated by the many different pathways that lead to severe disease and death and by the difficulty in determining the frequency with which these events occur.

Because of these uncertainties and knowledge gaps, establishing effective programs for public health control and personal protection is particularly important. Vaccines and drugs against circulating influenza viruses have been used for decades, but their efficacy in any future pandemic is difficult to predict because, with current knowledge, the causative agent of a future pandemic cannot be known in advance and may well be a novel virus whose susceptibility to existing drugs and vaccines has not been established. Important new technologies allow construction and pretesting of vaccines against all of the known influenza surface glycoproteins (16 hemagglutinins and 9 neuraminidases), although the likelihood that a new pandemic strain would be preventable by such vaccines cannot be known without an ability to predict its antigenic nature. Among additional strategies to overcome this limitation is development of "universal" vaccines based on antigens shared by many, and ideally all, influenza viruses.

The recent H5N1 epizootics in Southeast Asia serve as an important reminder of how few of the key determinants of pandemic influenza are really understood. If there is a single lesson to be learned from the articles in this issue, it is that, as expressed by contributor Anthony Fauci, more research is needed in many areas. We (Figures 1 and 2) do not know whether pandemic influenza will outpace the increasingly vigorous research to contain it. But the race is on, the stakes are high, and the world is nervously watching.

**Figure 1:**
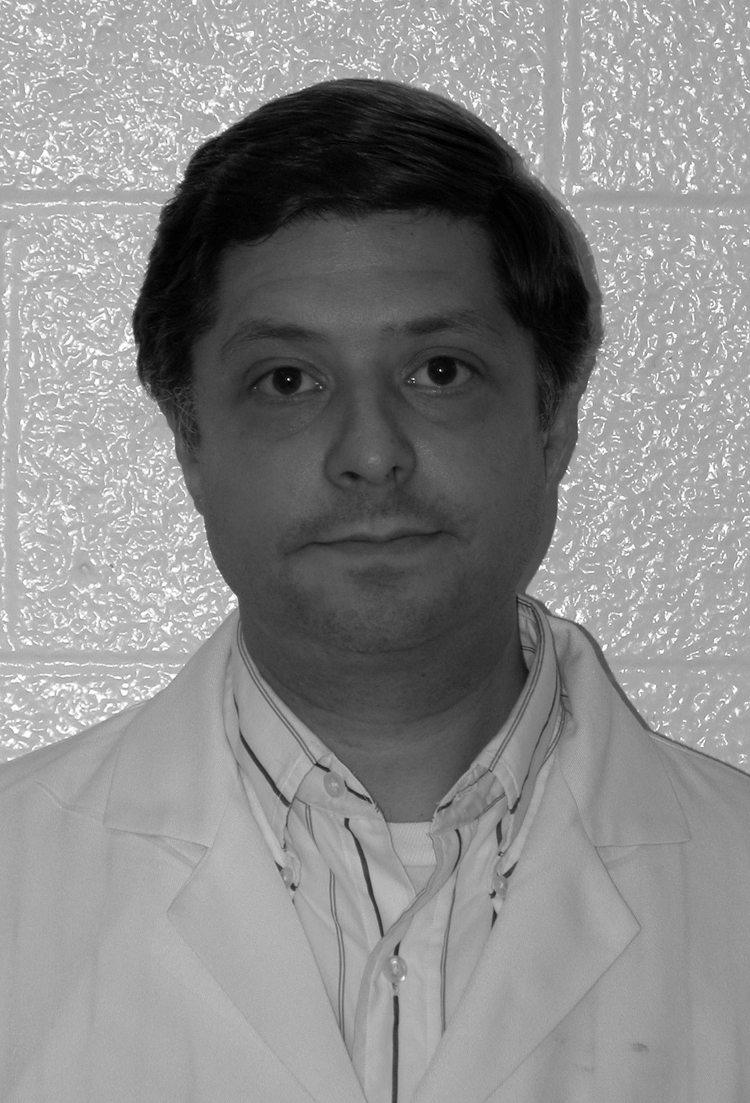
Photo of Jeffery K. Taubenberger. Dr Taubenberger is chair of the Department of Molecular Pathology at the Armed Forces Institute of Pathology in Rockville, Maryland. His clinical interest is in diagnostic molecular genetic pathology. His research interests include the molecular pathophysiology and evolution of influenza viruses.

**Figure 2:**
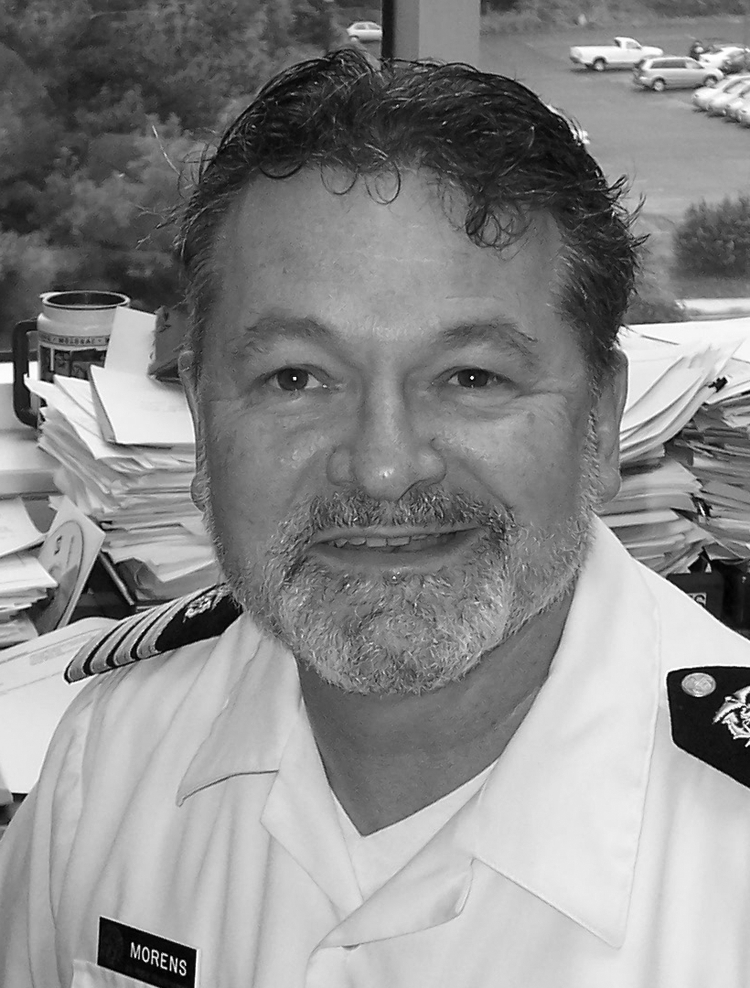
Photo of David M. Morens. Dr Morens is an epidemiologist with a long-standing interest in emerging infectious diseases, virology, tropical medicine, and medical history. He spent more than 6 years at the US Centers for Disease Control, followed by 17 years at the University of Hawaii. Since 1999, he has worked at the National Institute of Allergy and Infectious Diseases. He is an associate editor of Emerging Infectious Diseases

## References

[1] Fauci AS. Race against time. Nature. 2005;435:423–4. 10.1038/435423a 15917781

[2] Webby RJ, Webster RG. Are we ready for pandemic influenza? Science. 2003;302:1519–22. 10.1126/science.1090350 14645836

[3] Reid AH, Taubenberger JK, Fanning TG. Evidence of an absence: the genetic origins of the 1918 pandemic influenza virus. Nat Rev Microbiol. 2004;2:909–14. 10.1038/nrmicro1027 15494747 PMC7097663

[4] Taubenberger JK, Reid AH, Lourens RM, Wang R, Jin G, Fanning TG. Characterization of the 1918 influenza virus polymerase genes. Nature. 2005;437:889–93. 10.1038/nature04230 16208372

[5] Matrosovich MN, Matrosovich TY, Gray T, Roberts NA, Klenk HD. Human and avian influenza viruses target different cell types in cultures of human airway epithelium. Proc Natl Acad Sci U S A. 2004;101:4620–4. 10.1073/pnas.0308001101 15070767 PMC384796

[6] Shinya K, Hamm S, Hatta M, Ito H, Ito T, Kawaoka Y. PB2 amino acid at position 627 affects replicative efficiency, but not cell tropism, of Hong Kong H5N1 influenza A viruses in mice. Virology. 2004;320:258–66. 10.1016/j.virol.2003.11.030 15016548

[7] Glaser L, Stevens J, Zamarin D, Wilson IA, Garcia-Sastre A, Tumpey TM, A single amino acid substitution in 1918 influenza virus hemagglutinin changes receptor binding specificity. J Virol. 2005;79:11533–6. 10.1128/JVI.79.17.11533-11536.2005 16103207 PMC1193621

[8] Thompson WW, Shay DK, Weintraub E, Brammer L, Cox N, Anderson LJ, Mortality associated with influenza and respiratory syncytial virus in the United States. JAMA. 2003;289:179–86. 10.1001/jama.289.2.179 12517228

